# Characterization of EOP-1 reveals cell autonomous oscillations preceding somatic cell fusion in *Neurospora crassa*

**DOI:** 10.1371/journal.pgen.1012087

**Published:** 2026-03-31

**Authors:** Anne Geertje Oostlander, Marcel René Schumann, Lina Strzelczyk, Lucas Well, Ulrike Brandt, Marco Leiterholt, Stefan Jakschies, Tina Rietschel, Josef Wissing, Lothar Jänsch, André Fleißner

**Affiliations:** 1 Institute of Genetics, Technische Universität Braunschweig, Braunschweig, Germany; 2 Cellular Proteomics, Helmholtz Center for Infection Research, Braunschweig, Germany; 3 Braunschweig Integrated Centre of Systems Biology, Technische Universität Braunschweig, Braunschweig, Germany; University of Georgia, UNITED STATES OF AMERICA

## Abstract

Cell fusion is a fundamental process essential for the development and proliferation of eukaryotic organisms. In the ascomycete fungus *Neurospora crassa*, germinating spores undergo chemotropic interactions and fusion to merge into a supracellular unit, which gives rise to the mycelial colony. Within mature colonies, hyphal branches fuse to form anastomoses between leading hyphae, enhancing the overall connectivity of the mycelium. Both germling and hyphal fusion rely on the same molecular machinery. The MAP kinase MAK-2 and the fungal-specific protein SO have been identified as key regulators of these processes, and their alternating recruitment to the plasma membrane at interacting cell tips suggests a dialog-like cell communication mechanism involving dynamic switches between signal sending and receiving. However, the mechanisms that trigger the onset of this intercellular communication are still not understood. This study identifies EOP-1 as an interaction partner of the SO protein and functionally characterizes its role in cell communication and fusion. Deletion of the *eop-1* gene abolished germling fusion and chemotropic interactions, while live-cell imaging showed EOP-1 oscillating at interacting cell tips, coinciding with SO recruitment. Intriguingly, EOP-1 displayed a similar dynamic, oscillatory tip recruitment also in isolated, non-interacting germlings, setting it apart from previously characterized fusion factors in *N. crassa*. This observation suggests for the first time that spore germlings of *N. crassa* exhibit fusion related cell-autonomous oscillatory behavior and implicates EOP-1 in initiating intercellular communication. The oscillatory recruitment pattern of EOP-1 was dependent on the presence of SO, MAK-1, MAK-2, BEM1 and HAM-14 in the cell. Loss of EOP-1 strongly reduced MAK-1 phosphorylation, placing EOP-1 upstream of MAK-1 pathway activation. This work offers new insight into how genetically and developmentally identical cells initiate and coordinate their communication and mutual attraction.

## Author summary

In many filamentous fungi, colony establishment and growth involves somatic cell fusion. Genetically identical germinating spores merge to form a connected network that develops into the mycelium. Within this colony, additional fusions between hyphae create cross-connections that strengthen the overall network. Both processes depend on directional growth guided by a signaling system in which the two interacting partners alternately send and receive cues, establishing a kind of “cellular dialog.” The underlying mechanism involves dynamic cycles of assembly and disassembly of protein complexes at the growing tips. Until now, it has been unclear how this dialog begins. In this study, we characterized EOP-1, a protein that interacts with SO, a known factor probably involved in signal sending. Unexpectedly, EOP-1 was recruited to the tips of germlings even when no partner was present, showing rhythmic, oscillatory dynamics. This reveals that each cell has its own intrinsic signaling rhythm. When another germling comes into proximity, these rhythms become synchronized, and communication is established. We propose that even isolated cells emit pulsed signals in search of potential fusion partners. These findings provide new insight into how fungal cells initiate the communication that enables them to fuse, thereby ensuring successful colony formation and development.

## Introduction

Cell fusion is a fundamental part of the development of eukaryotic organisms, where two or more cells merge to form a single entity, facilitating the exchange of cytoplasmic and genetic material. This mechanism is integral to various physiological events, including fertilization in eukaryotic species, and muscle development or tissue regeneration in animals. The filamentous ascomycete fungus *Neurospora crassa* has emerged as a powerful model system for dissecting the molecular principles that govern cell fusion [1]. The growth and development of filamentous fungi involves multiple cell-cell fusion events during both vegetative and sexual stages [2]. In filamentous ascomycete fungi, vegetative growth involves two distinct somatic fusion events at different stages of colony development. Early in colony initiation, germinating spores undergo chemotropic interactions, grow toward one another, and fuse to form a supracellular unit - a process known as germling fusion. This initial fusion promotes efficient colony establishment by reducing competition for space and resources among genetically identical cells. In adult colonies, hyphae exhibit different growth behaviors depending on their position within the colony. While hyphal tips at the outer growth front actively avoid each other, hyphal branches within the older parts of the colony show mutual attraction and fusion (hyphal fusion), thereby forming secondary cross connections between major leading hyphae [3]. The resulting increasing interconnectivity of the colony enhances even nutrient distribution, signal transmission, and resilience to injuries [4]. The underlying molecular mechanisms governing germling and hyphal fusion largely overlap but are not completely identical [1,5,6]. Both processes follow the same sequence of cellular events: cell-cell recognition, directed growth of the two cells toward each other, physical contact, plasma membrane fusion, and finally cytoplasmic mixing. Various studies on *N. crassa* and other filamentous fungi have provided insights into the molecular mechanisms orchestrating these processes [7]. Cell-cell communication, directed growth and subsequent fusion rely on a conserved signaling network centered on two MAP kinase cascades, the STRIPAK signaling complex, and two NADPH oxidase complexes. This network interacts with additional conserved and fungal-specific factors, including the general cell polarity machinery, the cytoskeleton, secretory pathway regulators, and transcription factors, adding up to more than 80 proteins identified so far [1]. The two MAP kinase cascades involved are the MAK-1 (NCU09842) pathway, homologous to the yeast cell wall integrity pathway, and the MAK-2 (NCU02393) pathway, homologous to the yeast pheromone response pathway. Targets of the two MAP kinases include transcription factors, other kinases and cytoskeletal elements. Gene deletion mutants in both pathways exhibit a pleiotropic phenotype, affecting colony morphology, aerial hyphal growth, sporulation, growth rate, and sexual development [8–10].

Another early-identified factor in cell fusion is the SO protein (Soft) (NCU02794, also called HAM-1). SO mutants exhibit pleiotropic phenotypes, are affected in vegetative growth and sexual reproduction, and are deficient in germling and hyphal fusion, underscoring the importance of SO in various cellular processes. SO is exclusively found in filamentous ascomycete fungi, and its precise molecular function is not fully understood [11]. The SO homolog in the closely related fungus *Sordaria macrospora* (PRO40) functions as a scaffold linking the MAK-1 MAP kinase module to its upstream activator protein kinase C [12]. In *N. crassa*, SO physically interacts with MIK-1 (NCU02234) and MEK-1 (NCU06419), but not MAK-1 [13]. During the interaction of fusing *N. crassa* spore germlings or hyphae, SO exhibits characteristic subcellular spatial and temporal dynamics. The protein initially resides in the cytoplasm of germinating spores. However, when two germlings establish an interaction, SO is recruited to the plasma membrane of the growing cell tip of one of the germlings. After 4–6 minutes, the protein is released from the tip and is simultaneously recruited to the membrane of the partner cell. This dynamic continues in an oscillating manner coordinated between the two cells in exact antiphase until both cells establish contact and the protein accumulates at the contact point [14]. This localization appears to be independent of the proposed SO function as a MAK-1 pathway scaffold, since these factors do not co-localize during directed growth [13].

Intriguingly, a comparable oscillating recruitment pattern to the cell tips is also observed for the MAP kinase MAK-2, however in exact antiphase to SO. When SO is recruited to the membrane of one germling, MAK-2 is recruited to the membrane of the other cell. The two proteins are never simultaneously recruited to the same tip. This observation led to the hypothesis that the genetically and developmentally identical cells alternate between signal emission and signal reception. Given the well-established role of MAP kinases in processing external signals, the cell with MAK-2 at the tip is thought to be in the signal reception mode, while the cell with SO at the tip is assumed to be in the signal-sending mode [14]. This coordinated, alternating membrane recruitment of the two signaling complexes could enable the two cells to establish a bi-directional communication based on a single signal/receptor pair [14,15].

This model relies on finely tuned feedback loops, with MAK-2 potentially playing a central role [15]. When the cell is in a signal-receiving state, the MAP kinase cascade, consisting of NRC-1 (MAPKKK, NCU06182), MEK-2 (MAPKK, NCU04612), and MAK-2 (MAPK) assembles at the plasma membrane together with the scaffold protein HAM-5 (NCU01789). This recruitment amplifies MAK-2 activation through a positive feedback mechanism. HAM-5 is progressively phosphorylated by MAK-2 and other kinases, ultimately resulting in the disassembly of the complex and the termination of the signal-receiving phase [16]. Activated MAK-2 also translocates to the nucleus, where it regulates transcription of fusion-related genes [17]. In parallel, MAK-2 activity also indirectly influences the nuclear import of the second MAP kinase MAK-1 through activation of MOB-3 (NCU07674), a protein of the STRIPAK complex [16,18]. Additionally, the presence of activated MAK-2 at the plasma membrane prohibits simultaneous recruitment of the SO protein, probably contributing to the coordinated, alternating membrane recruitment of the two factors [19]. Although SO and MAK-2 never co-localize, the proper subcellular dynamics of SO depend on MAK-2 activity [14].

The mathematical modeling of the interaction further supports the hypothesis that excitable behavior might mechanistically account for the coordinated behavior of the cells. In this process, a pulse-like signal from one cell triggers an excitation reaction in the second cell, culminating in the emission of a signal from the second cell. The model points to a role for vesicles in the signaling process, with MAK-2 regulating both their docking and fusion [15]. Storing the chemoattractant in vesicles enables a rapid, burst-like release upon vesicle fusion with the plasma membrane, rather than a continuous secretion. This mechanism ensures a fast and coordinated signal output, similar to neurotransmitter release at synapses, where vesicle-mediated exocytosis allows precise, pulse-like transmission [15].

Despite these insights, key questions remain. The identity and even the existence of the proposed vesicles have not yet been experimentally confirmed. Neither the signal nor its receptor involved in the signaling process have been identified. Furthermore, the mechanism that initiates signaling between the fusing cells remains unknown.

According to the current model of the signaling process, SO is involved in signal transmission at the germling tip, although its precise molecular function remains unknown. As such, SO serves as a valuable entry point for investigating the unresolved aspects of this mechanism. This study aimed to gain deeper insight into SO function by identifying and characterizing interacting proteins. Using co-immunoprecipitation, we found that SO interacts with EOP-1 (NCU04645), a factor previously linked to germling fusion and regulated by the transcription factor ADV-1 (NCU07392) [10]. By characterization of the gene knock out mutant we also identified roles in hyphal fusion and sexual development. Via live cell imaging we demonstrate that EOP-1 and SO exhibit comparable subcellular dynamics during directed growth of the fusion cells. In contrast to all other known factors involved in cell fusion in *N. crassa*, EOP-1 oscillates already to the membrane in individual, non-interacting spore germlings, indicating that each of these cells possesses a cell autonomous internal rhythm. Phenotype characterization and biochemical analysis revealed a potential function of EOP-1 in activation of the MAK-1 pathway. Together, our data implicate EOP-1 as a novel regulator of MAK-1 signaling and expand the known network controlling fungal cell fusion.

## Results

### EOP-1 is an interaction partner of SO

**Fig 1:**
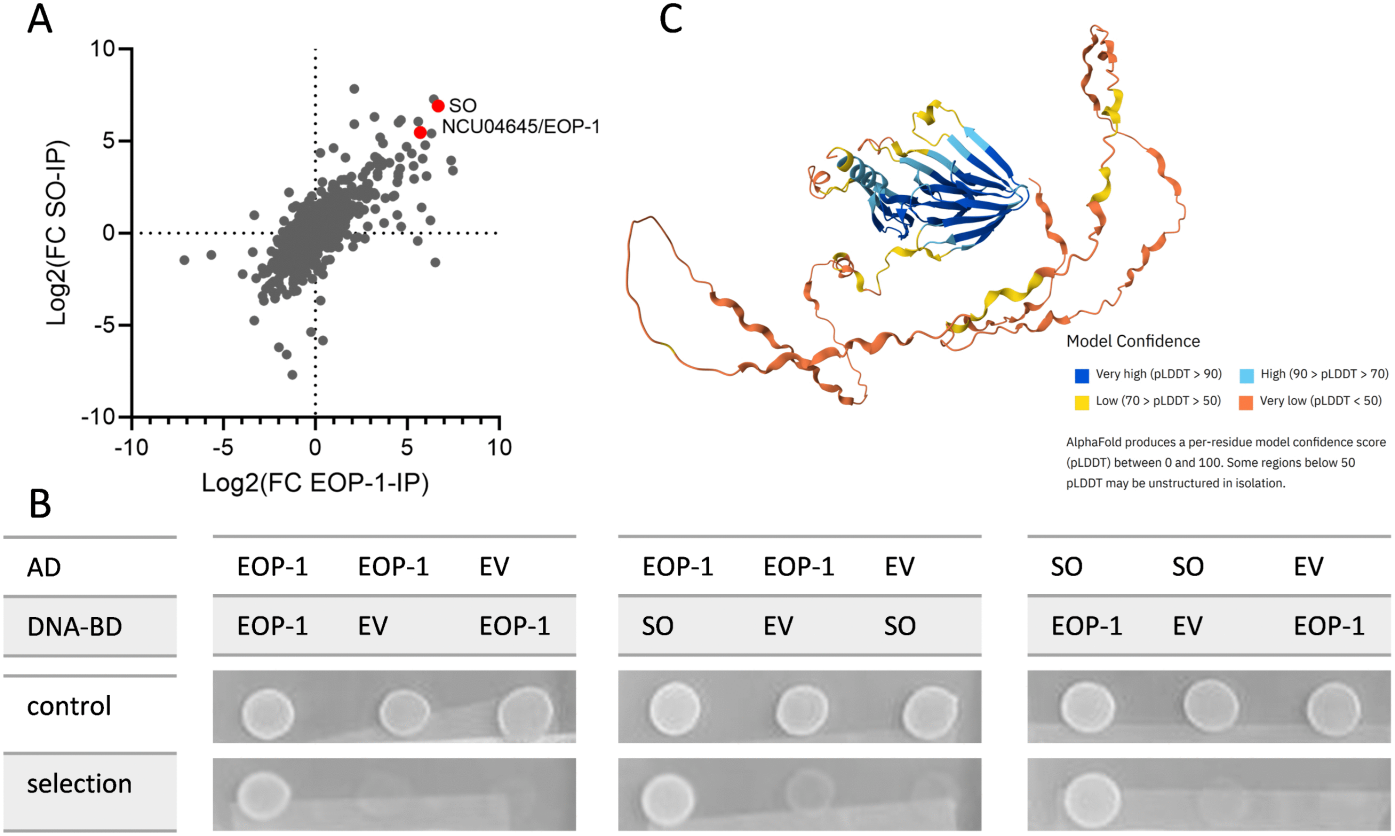
EOP-1 was identified as an interaction partner of SO through proteomic and Y2H analyses. **(A)** Scatter plot of MS data depicting the fold-change enrichment of eluate fractions compared to the wash in SO-CoIP (y-axis) and SIP-1-CoIP (x-axis) **(B)** Y2H assay demonstrating interactions of EOP-1. Yeast co-expressing BD-EOP-1 with AD-SO or AD-EOP-1 with BD-SO grew on selective media (SD-Leu-Trp-Ade-His), indicating interactions between SO and EOP-1, as well as EOP-1 self-interaction. Negative controls (BD-EOP-1 or AD-SO co-transformed with empty vectors (EV)) showed no growth. (n = 3) **(C)** AlphaFold 2 structural model of EOP-1 (NCU04645) showing a disordered N-terminal region and a well-folded C-terminal domain belonging to the AIM24 family.

To further investigate the molecular function of SO and the mechanism of signal sending during cell-cell communication, preliminary experiments were conducted to identify potential interaction partners. Initial immunoprecipitation experiments searching for potential binding partners of SO in *N. crassa* hinted on its interaction with the protein NCU04645 (DUF124 domain-containing protein) (S1 Table). To confirm these results, Co-immunoprecipitations (Co-IPs) were performed using both SO and NCU04645 as baits. IP-eluate and wash fractions were separated by SDS-PAGE, followed by in-gel digestion, protein purification and mass spectrometry (MS) analysis. Across all fractions, 800 proteins were stably identified. Both SO and NCU04645 were highly enriched in the eluate fractions of both Co-IPs (S2 Table). A comparative analysis of fold change enrichment between eluate and wash confirmed strong mutual enrichment, supporting a robust interaction between SO and NCU04645 (Fig 1A). NCU04645, which has been identified as factor essential for germling fusion in *N. crassa* [10] will hereafter be referred to as Early Oscillating Protein 1 (EOP-1).

To validate the interaction between EOP-1 and SO suggested by the initial pulldown assay, a yeast two-hybrid (Y2H) assay was performed (Fig 1B). This assay confirmed a direct interaction between SO and EOP-1, supporting the MS findings. Notably, EOP-1 also demonstrated the ability to form homomultimers, as evidenced by its interaction with itself in the Y2H assay.

Since the role and function of this protein remain, however, unknown, we decided to undertake a detailed functional characterization. The *eop-1* open reading frame, located on Chromosome V, spans 3201 base pairs. Sequencing of its cDNA confirmed the presence of six exons and five introns. The encoded protein, EOP-1, consists of 445 amino acids. Its N-terminal region (amino acids 1–230) is rich in proline and glutamine. Protein structure prediction indicated that this section of the protein lacks a defined structure, while the C-terminal region (amino acids 231–429) includes an AIM24-like domain (Fig 1C). BLAST analysis at Mycocosm showed that EOP-1 paralogues and/or orthologues are common in fungi, while the AIM24-like domain is present across all three domains of life in proteins with diverse functions.

### EOP-1 is essential for cell-cell communication

**Fig 2:**
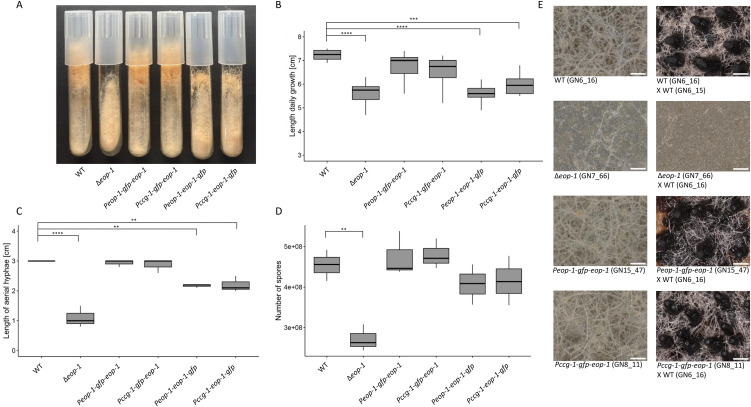
Loss of EOP-1 reduces hyphal growth, aerial hyphae length, and sporulation in *N. crassa.* **(A)** Colony morphology of wild type (WT), ∆*eop-1*, and complemented strains (∆*eop-1 Peop-1-gfp-eop-1*, ∆*eop-1 Pccg-1-gfp-eop-1*, ∆*eop-1 Peop-1-eop-1-gfp*, ∆*eop-1 Pccg-1-eop-1-gfp*) grown on VMM slants. Cultures were incubated for 2 days at 30°C in darkness, followed by 3 days at room temperature with a day-night cycle. **(B)** Daily linear growth measured in race tube assays (n = 9). **(C)** Aerial hyphal length after 7 days of incubation at 30°C in darkness (n = 3). **(D)** Sporulation quantified as spore count after 4 days at 30°C in darkness, followed by 3 days at room temperature with a day-night cycle (n = 3). **p ≤ 0.01/***p ≤ 0.001/****p ≤ 0.0001 **(E)** Female sexual development on Westergaard’s medium. Protoperithecia formation was assessed after 7 days of incubation on Westergaard’s medium at 26°C under day light (images on the left, n = 3). ∆eop-1 mutants formed very few protoperithecia while the WT and the complemented strains ∆*eop-1 Peop-1-gfp-eop-1* and ∆*eop-1 Pccg-1-gfp-eop-1* showed normal protoperithecia formation. Fertility tests showed the WT and the complemented strains ∆*eop-1 Peop-1-gfp-eop-1* and ∆*eop-1 Pccg-1-gfp-eop-1* developed normal perithecia while the ∆eop-1 mutant was sterile as female mating partner (images on the right, n = 3). The strain used as female is listed first.

The earlier, preliminary characterization of the ∆*eop-1* mutant strain revealed a defect in germling interactions related to cell-cell fusion. The macroscopic phenotype include decreased hyphal extension rates and shortened aerial hyphae similar to the appearance of the ∆*so* strain (Fig 2A) [10,11]. To validate the preliminary data and confirm that the macroscopic phenotype of the ∆*eop-1* mutant is indeed due to the loss of *eop-1*, we compared the mutant with both the wild-type isolate and a ∆*eop-1* strain in which the *eop-1* gene was reintroduced (∆*eop-1 Peop-1-gfp-eop-1*). As a result, the preliminary data were confirmed and the wild-type phenotype was fully restored in the complemented strain (Fig 2B-2D). In addition, we identified a sporulation defect of the *eop-1* mutant, indicated by highly reduced spore formation (Fig 2D).

**Fig 3:**
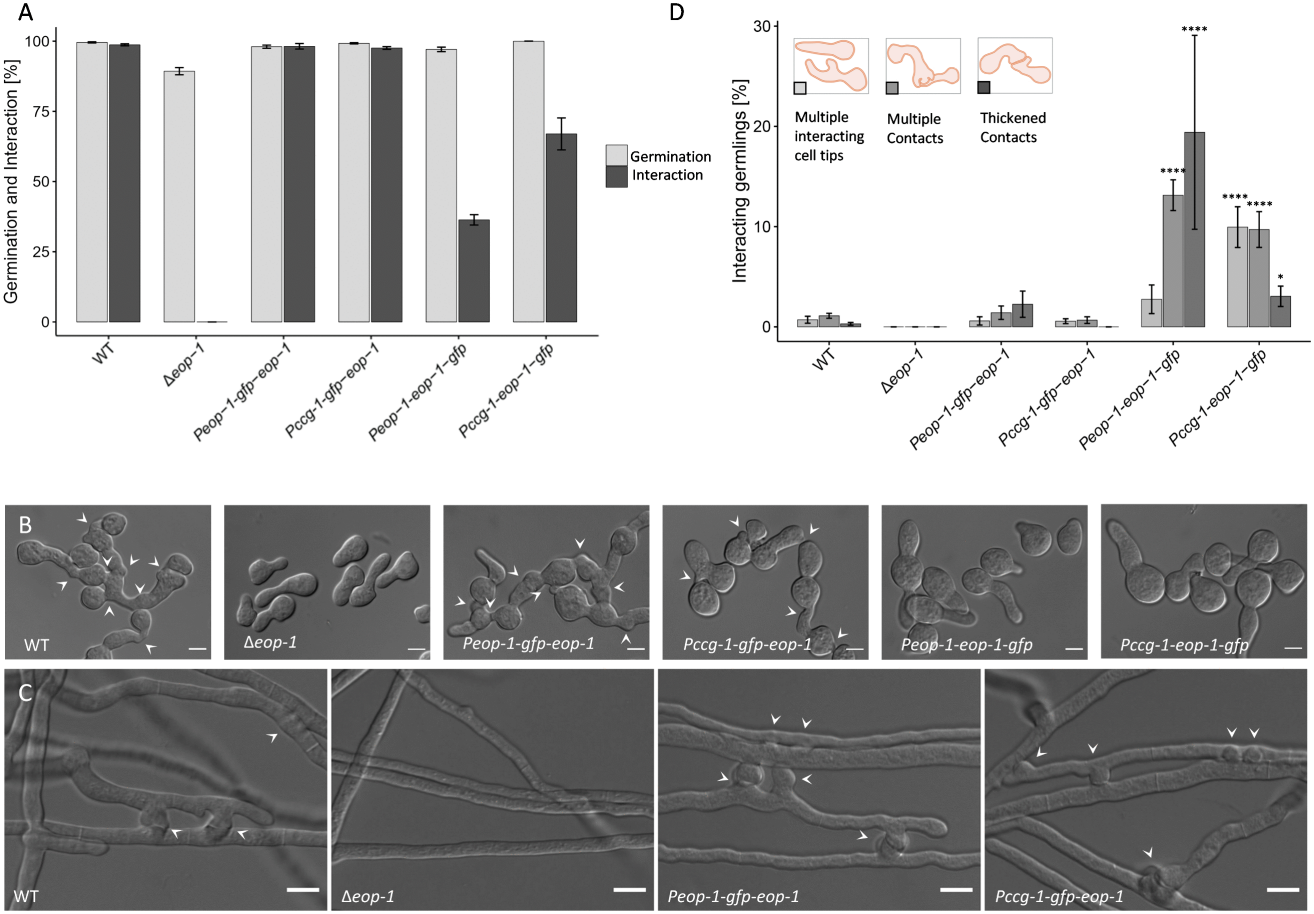
EOP-1 is essential for germling and hyphal fusion in N. crassa. **(A)** Quantification of germination and germling interaction rates in WT, ∆*eop-1*, and the complemented strains (∆*eop-1 Peop-1-gfp-eop-1*, ∆*eop-1 Pccg-1-gfp-eop-1*, ∆*eop-1 Peop-1-eop-1-gfp*, ∆*eop-1 Pccg-1-eop-1-gfp*), showing complete loss of interaction in ∆*eop-1* (n = 900). **(B)** Microscopic images of germinating conidia from WT, ∆*eop-1*, and the complemented strains. WT and complemented strains ∆*eop-1 Peop-1-gfp-eop-1* and ∆*eop-1 Pccg-1-gfp-eop-1* show normal germling interaction (arrow heads), while ∆*eop-1 Peop-1-eop-1-gfp*, ∆*eop-1 Pccg-1-eop-1-gfp* germlings show reduced interaction and ∆*eop-1* germlings fail to orient growth toward each other. **(C)** Microscopic examination of 2-day-old colonies reveals abundant hyphal anastomoses in WT and complemented strains ∆*eop-1 Peop-1-gfp-eop-1* and ∆*eop-1 Pccg-1-gfp-eop-1* (arrow heads), but complete absence in ∆*eop-1* colonies. (n = 10) **(D)** Quantification of atypical germling interaction behavior, including multiple interacting cell tips, multiple contacts, and thickened contact sites. Mutants expressing *Pccg-1-eop-1-gfp* or *Peop-1-eop-1-gfp* showed significantly higher occurrences compared to WT (n = 900). *p ≤ 0.05/ ****p ≤ 0.0001. All scale bars: 5 µm.

To further confirm and investigate the impact of the ∆*eop-1* mutation on the process of cell fusion, we assessed germinating conidia from the wild type, ∆*eop-1*, and the complemented strains (∆*eop-1 Peop-1-gfp-eop-1*). While the wild-type and complemented strains exhibited typical and comparable interaction rates, germlings of the *∆eop-1* strain completely failed to orient their growth towards each other (Fig 3A and 3B).

Most proteins involved in germling fusion in *N. crassa* also play a role in hyphal fusion within the mature colony. To determine if this process also requires EOP-1, we microscopically examined 2-day-old colonies of the wild type, the *∆eop-1* mutant, and the complemented strains (∆*eop-1 Peop-1-gfp-eop-1*). Hyphal anastomoses were consistently observed in the inner colony regions of the wild type and the complemented strain, but were fully absent in the *∆eop-1* mutant (Fig 3C).

### EOP-1 is essential for fertilization

Previous studies have shown that the ∆*so* mutant is sterile as female mating partners but can still initiate fruiting body development by the formation of protoperithecia. To investigate a potential role of EOP-1 in sexual development, we analyzed protoperithecia formation on Westergaard’s medium in wild-type and ∆*eop-1* mutants of both mating types. The ∆*eop*-*1* mutants produced significantly fewer protoperithecia compared to the wild type, suggesting a role for EOP-1 in early protoperithecia development (S1 Fig). Fertilization assays further demonstrated that ∆*eop-1* mutants are sterile as female mating partners but fertile as male mating partners, resulting in normal ascospore formation.

To proof whether the female sterility phenotype is indeed caused by the loss of eop-1, we analyzed female sexual development on Westergaard’s medium in wild type, ∆*eop-1*, and the two complemented strains ∆*eop-1 Peop-1-gfp-eop-1*, and ∆*eop-1 Pccg-1-gfp-eop-1* (Fig 2E). While the ∆*eop-1* mutant formed fewer protoperithecia, which also failed to mature into perithecia after fertilization, the two complemented strains ∆*eop-1 Peop-1-gfp-eop-1* and ∆*eop-1 Pccg-1-gfp-eop-1* showed development comparable to the wild type. In summary, the macroscopic and microscopic ∆*eop-1* mutant phenotype is highly reminiscent of strains lacking the *so* gene, further indicating that these two interacting proteins functions together in the growth and development of *N. crassa*.

### GFP-EOP-1 shows dynamic tip localization during germling interaction

SO is recruited to the tips of interacting germlings in an oscillatory manner. Since EOP-1 is essential for cell-cell interactions and interacts with SO, we hypothesized that EOP-1 might show similar subcellular dynamics. To test this hypothesis, we performed fluorescence microscopy on the complemented strain, which expresses N-terminally GFP-tagged EOP-1 under control of the native promoter (∆*eop-1 Peop-1-gfp-eop-1*). However, the fluorescence signal in this strain was too weak for reliable localization. Therefore, we used two alternative approaches: employing an overexpression promoter and/or shifting the GFP tag to the C-terminus of the protein. The resulting strains ∆*eop-1 Pccg-1-gfp-eop-1*, ∆*eop-1 Peop-1-eop-1-gfp* and ∆*eop-1 Pccg-1-eop-1-gfp* were first assessed for complementation of the macroscopic and microscopic deletion phenotypes (Fig 2). Only strain ∆*eop-1 Pccg-1-gfp-eop-1* exhibited a phenotype comparable to that of the wild type. Expression of the C-terminally tagged EOP-1 (strains ∆*eop-1 Peop-1-eop-1-gfp* and ∆*eop-1 Pccg-1-eop-1-gfp*) resulted in an intermediate phenotype between the deletion mutant and wild type. Thus, ∆*eop-1 Pccg-1-gfp-eop-1* was chosen for fluorescence microscopy to study EOP-1 localization.

**Fig 4:**
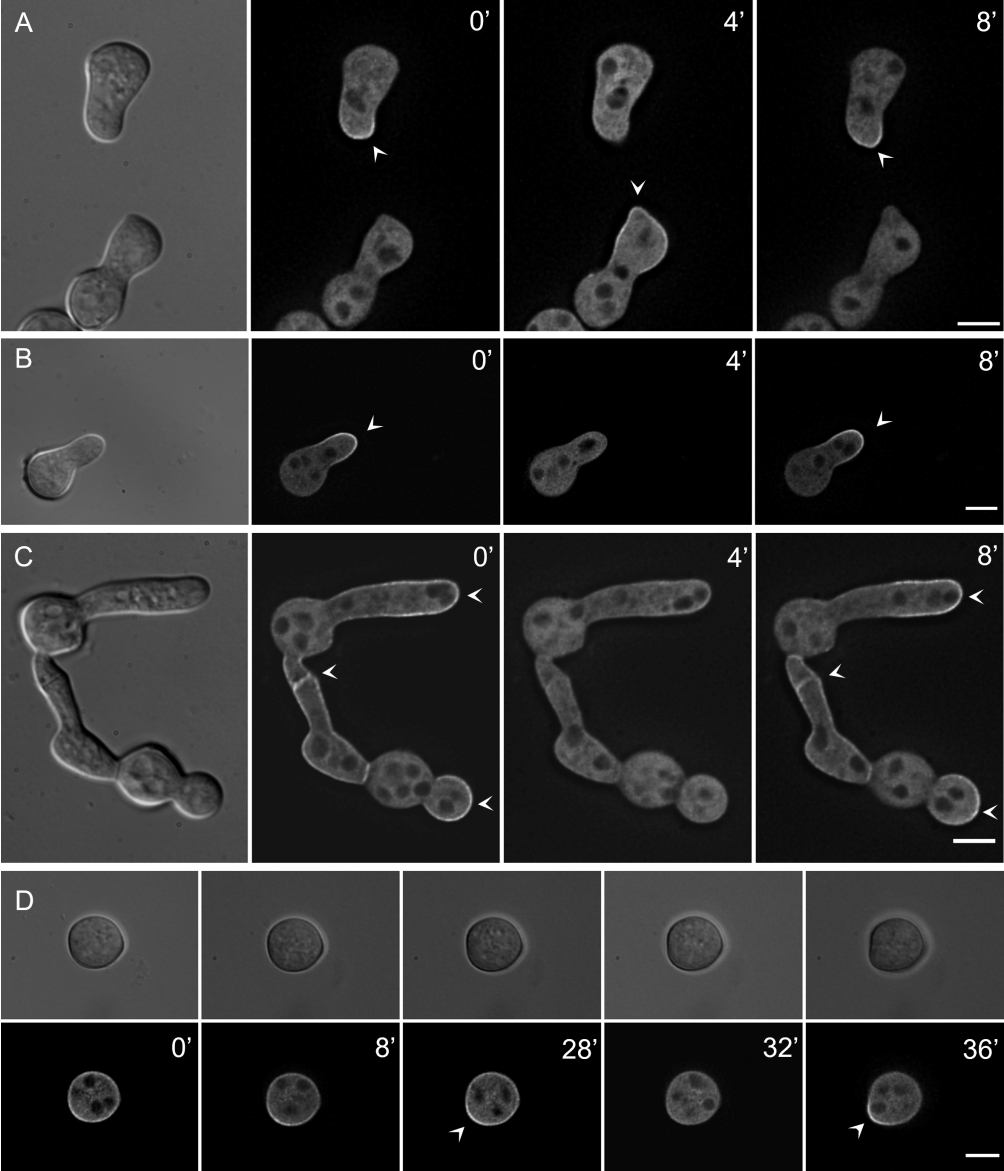
Dynamic localization of GFP-EOP-1 in germinating and interacting germlings of strain∆*eop-1 Pccg-1-gfp-eop-1.* **(A)** GFP-EOP-1 localizes to the cytoplasm and oscillates at ~8-minute intervals to the plasma membranes of interacting germlings (arrow heads), partner cells alternating in the recruitment of the protein during directed growth (n = 5). **(B)** Time-lapse microscopy shows consistent GFP-EOP-1 oscillation dynamics in individual cell (arrow heads) (n = 10), indicating cell-autonomous and coordinated oscillation, unique to GFP-EOP-1. **(C)** Post-fusion recruitment of GFP-EOP-1. After fusion, GFP-EOP-1 oscillates simultaneously at all growing tips in the young network (arrow heads). (n = 9) **(D)** During spore germination, GFP-EOP-1 initially localizes to the cytoplasm with transient membrane association (arrow heads). Prior to germination, GFP-EOP-1 accumulates at a specific membrane site, marking the future germ tube emergence point. Oscillation begins as the germ tube forms. (n = 4) All scale bars: 5 µm.

To observe GFP-EOP-1 localization in germlings, spores of the ∆*eop-1 Pccg-1-gfp-eop-1* strain were incubated on VMM at 30°C for 3.5 hours, followed by fluorescence microscopy (Fig 4). GFP-EOP-1 was detected in the cytoplasm and accumulated dynamically at the plasma membrane of the growing cell tips in interacting germlings. This membrane association oscillated with a ca. 8-minute phase (Fig 4A). The two interacting germlings coordinated their behavior such that one partner recruited GFP-EOP-1 to its tip while the protein remained cytosolic in the second partner. After about 4 minutes, GFP-EOP-1 disappeared from the tip of the first germling, and the second germling simultaneously recruited GFP-EOP-1 to its tip. This oscillatory recruitment continued during directed growth of the cells towards each other. The spatial and temporal dynamics of this subcellular localization is very highly reminiscent to the localization pattern of SO in interacting germlings.

After physical contact was established, GFP-EOP-1 continued to oscillate at the contact point (Fig 4C). Subsequently, the newly formed cellular unit began to recruit EOP-1 to its newly growing tip(s). When multiple growing tips emerged, they recruited and released GFP-EOP-1 simultaneously, indicating that the entire unit oscillates in phase (Fig 4C). Thus, the cells transition from an anti-phase dynamic before fusion to synchronous behavior after cell merger.

### GFP-EOP-1 recruitment oscillates in non-interacting germlings

During live-cell imaging, we frequently observed GFP-EOP-1 tip association in individual, non-interacting germlings. To determine if this recruitment follows the same spatial and temporal dynamics as in interacting germlings, we conducted time-lapse microscopy on both individual and interacting cells. The GFP-EOP-1 oscillation frequency was consistent between interacting and non-interacting germlings (Fig 4B), indicating that germlings exhibit both cell-autonomous EOP-1 oscillation and coordinated oscillation in interacting cell pairs (Fig 4A and 4B). A similar cell autonomous dynamic has not been observed for any other protein mediating the cell-cell dialog signaling mechanism in *N. crassa*, including the SO protein. This observation suggests that EOP-1 may play a role in the initial stages of initiating cell-cell interactions. To determine when GFP-EOP-1 recruitment starts, the localization of GFP-EOP-1 during spore germination was assessed. In un-germinated spores, GFP-EOP-1 is in the cytoplasm and exhibits some membrane association without a clear, stable pattern (Fig 4D). However, just before germination, GFP-EOP-1 distinctly accumulates at a specific membrane spot. This accumulation increases over time, with the spore eventually forming a germ tube at the GFP-EOP-1 recruitment site. The oscillation of the protein begins as soon as the germ tube starts to emerge.

### GFP-EOP-1 and SO-dsRed localize at the same cell tip of interacting germlings

**Fig 5:**
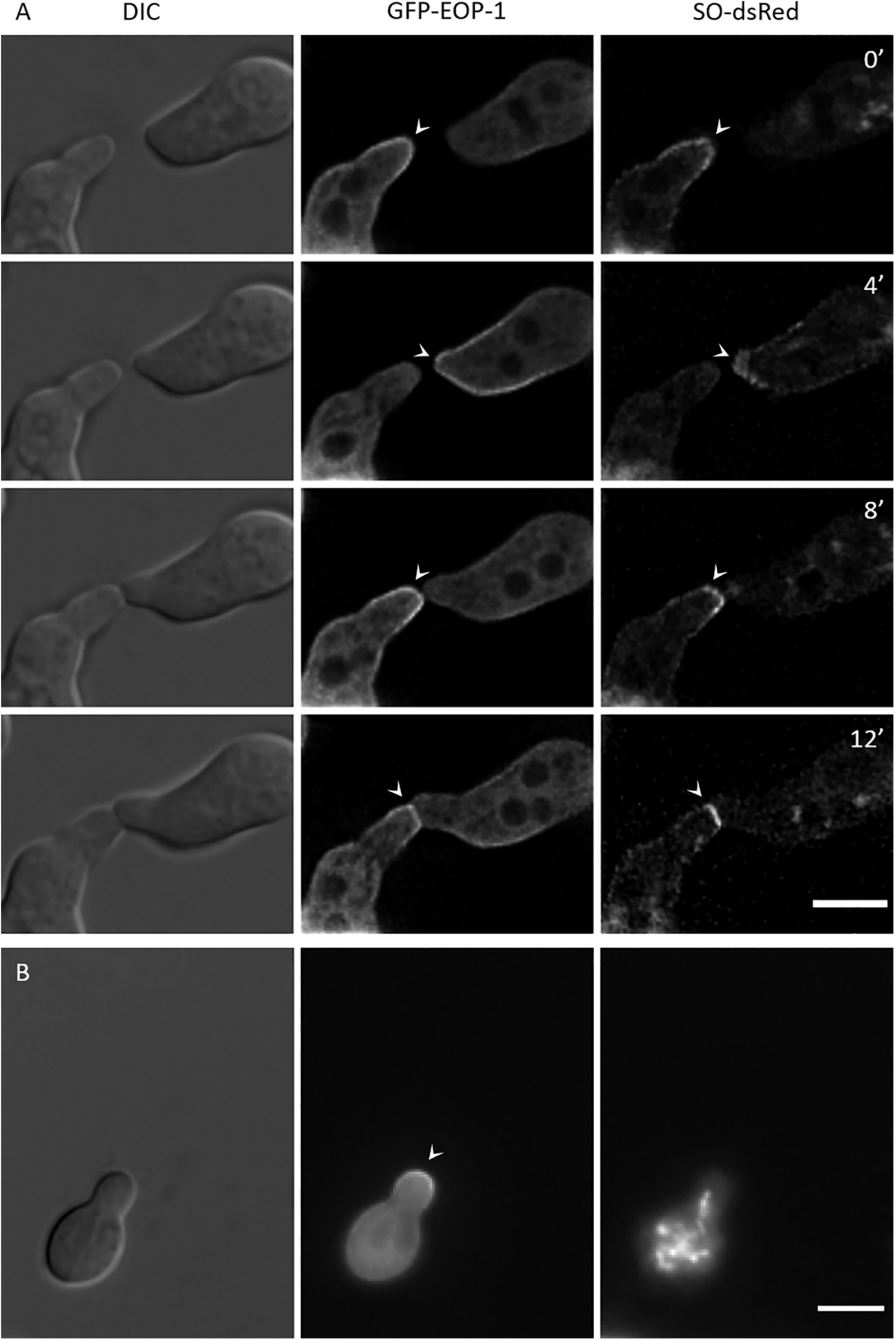
Co-localization of GFP-EOP-1 and SO-dsRed in germlings of the strain *gfp-eop-1 so-dsRed.* **(A)** GFP-EOP-1 and SO-dsRed co-localize at tips of interacting germlings (arrow heads) in 4-minute intervals, until physical contact is made and both proteins accumulate at the contact site. (n = 15) **(B)** In individual germlings without partners, GFP-EOP-1 is recruited to tips (arrow heads), but SO-dsRed remains cytoplasmatic (n = 20). All scale bars: 5 µm.

Based on our previous findings that EOP-1 and SO interact, we hypothesized that both proteins are simultaneously recruited to germling tips. To test this notion, we constructed a strain co-expressing GFP-EOP-1 and SO-dsRed using a novel co-expression strategy. Both expression constructs were cloned into a single plasmid, which integrates at the *his-3* locus, avoiding the need for heterokaryons, which are commonly used in co-expression studies in *N. crassa*. In the resulting strain *gfp-eop-1 so-dsRed*, both proteins co-localized at the tips of interacting germlings with identical temporal dynamics until physical contact was established (Fig 5A). In contrast, individual germlings without interaction partners that recruited GFP-EOP-1 to their germ tube tips did not show SO-dsRed recruitment (Fig 5B). These results further support the hypothesis that EOP-1 and SO function together following the initiation of communication.

### Coordination of oscillatory behavior between cells

**Fig 6:**
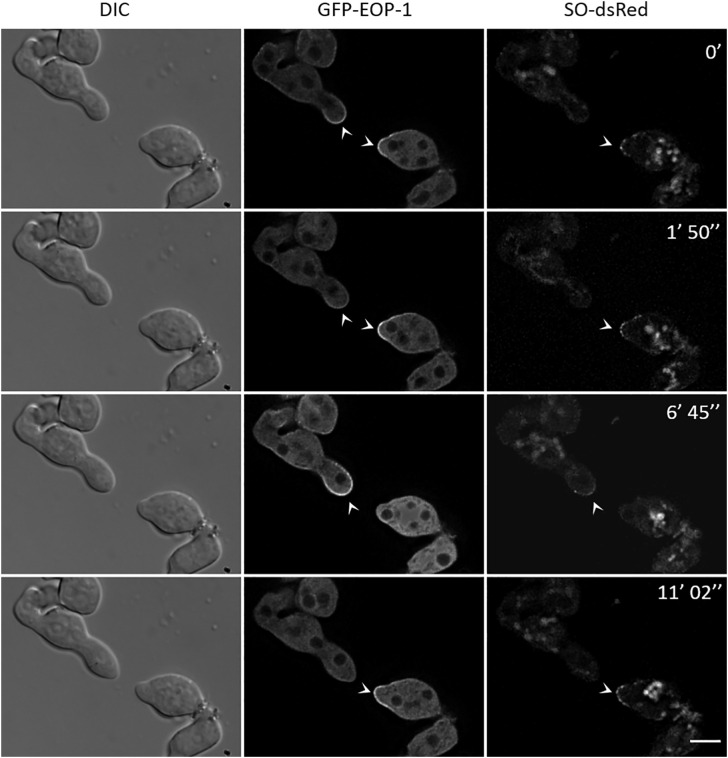
Coordination of GFP-EOP-1 and SO-dsRed oscillation during germling interaction. Time-lapse imaging shows the transition from uncoordinated to coordinated oscillation of GFP-EOP-1 and SO-dsRed in an interacting germling pair of the strain *gfp-eop-1 so-dsRed*. Initially, GFP-EOP-1 is recruited to both tips, but SO-dsRed localizes to only one (arrow heads). By 6:45 min, the germlings alternate recruitment of both proteins to their tips in synchronized 4-minute intervals. All scale bars: 5 µm.

To gain a deeper understanding of the initiation of communication and the required coordination of the oscillatory protein recruitment to interacting cell tips, we tracked the localization of GFP-EOP-1 and SO-dsRed in a germling pair of the strain *gfp-eop-1 so-dsRed* (Fig 6). At the initial timepoint, both facing cells showed GFP-EOP-1 recruitment to their tips, indicating that the anti-phase oscillatory pattern had not yet been established. While in the first germling, SO-dsRed was absent from the tip, both proteins co-localized in the second germling. After 1 minute and 50 seconds, GFP-EOP-1 was released from the first cell’s tip slightly earlier then from the second cell, which continued to accumulate GFP-EOP-1 and SO-dsRed. This event was followed by apparent coordination of protein oscillation between the two cells. At 6 minutes and 45 seconds, the first cell recruited GFP-EOP-1 and SO-dsRed to its tip, while the second cell released both proteins into the cytoplasm. Approximately 4 minutes later, at 11 minutes and 2 seconds, the second germling recruited both proteins as they were released from the first germling’s tip. This alternating pattern indicated that the cells had entered a coordinated oscillation with ~8-minute intervals, consistent with prior observations of interacting cells. These results demonstrate that two germlings transition from initially uncoordinated to coordinated recruitment of the proteins to the plasma membrane by adjusting the timing of their individual oscillations.

### EOP-1 tip localization is not related to general polar growth

EOP-1 was consistently found oscillating at the polarized tip of germlings from the onset of germination, in both single and interacting germlings. This raised the question if EOP-1 plays not only a role in fusion-related communication and directed growth but also in general polar growth. In contrast to spore germlings, hyphae in mature mycelial colonies allow a clear distinction between general polar growth and directed growth. In the inner parts of the colony, hyphae grow toward each other in a directed manner to fuse, while at the outer colony edge, hyphae avoid one another and represent exclusively polar growing structures.

**Fig 7:**
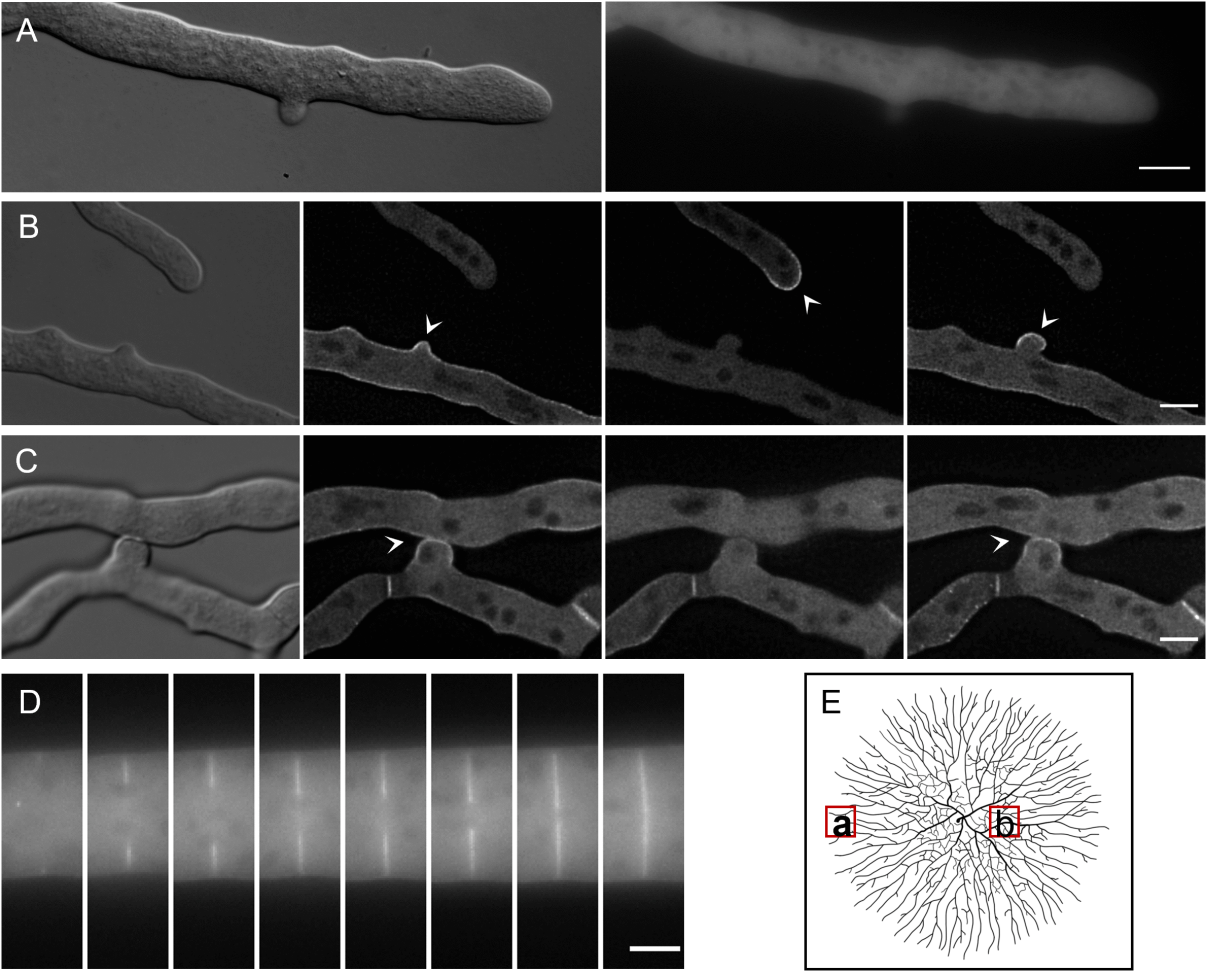
Localization of GFP-EOP-1 in hyphae during polar and directed growth in the strain∆*eop-1 Pccg-1-gfp-eop-1*. (A) At the colony edge, hyphae exhibit exclusively polar growth. GFP-EOP-1 localizes to the cytoplasm with no recruitment to hyphal tips (n = 20). (B, C) Inside the colony, during directed growth and fusion, GFP-EOP-1 oscillates between interacting hyphal tips with a 4-minute interval (arrow heads). After contact, GFP-EOP-1 localizes at the fusion site (arrow heads) (n = 6). (D) GFP-EOP-1 is also observed at hyphal septa during and after formation (n = 8). (E) Schematic representation of sampling locations for microscopy, indicating colony edge hyphae analyzed for polar growth (a), and internal hyphae for directed growth and fusion (b). (changed after Buller *1958)* Scale bar (A): 10 µm, Scale bar (B, C, D): 5 µm.

To test if the function of EOP-1 is also related to polar growth, we determined the localization of GFP-EOP-1 at hyphal tips at the edge of the mycelial colony and compared it to interacting hyphae in the colony´s center. GFP-EOP-1 predominantly localized in the cytoplasm of hyphae at the colony edge, with no observable recruitment to the membrane of hyphal tips, suggesting that EOP-1 does not contribute to general polar growth (Fig 7A). In contrast, in hyphae undergoing directed growth and subsequent fusion in the inner part of the colony, GFP-EOP-1 exhibited the typical oscillatory membrane recruitment observed during germling interaction and after cell contact (Fig 7B and 7C).

This observation suggests that EOP-1 does not contribute to general polar growth but has a specific function in fusion-related directed growth. In the course of analyzing EOP-1 dynamics in hyphae, we also noticed that GFP-EOP-1 localizes to hyphal septa during and after their formation (Fig 7D). However, the Δ*eop-1* mutant displayed normal septation, indicating that EOP-1 is dispensable for this process.

### A deletion of SO affects EOP-1 recruitment

**Fig 8:**
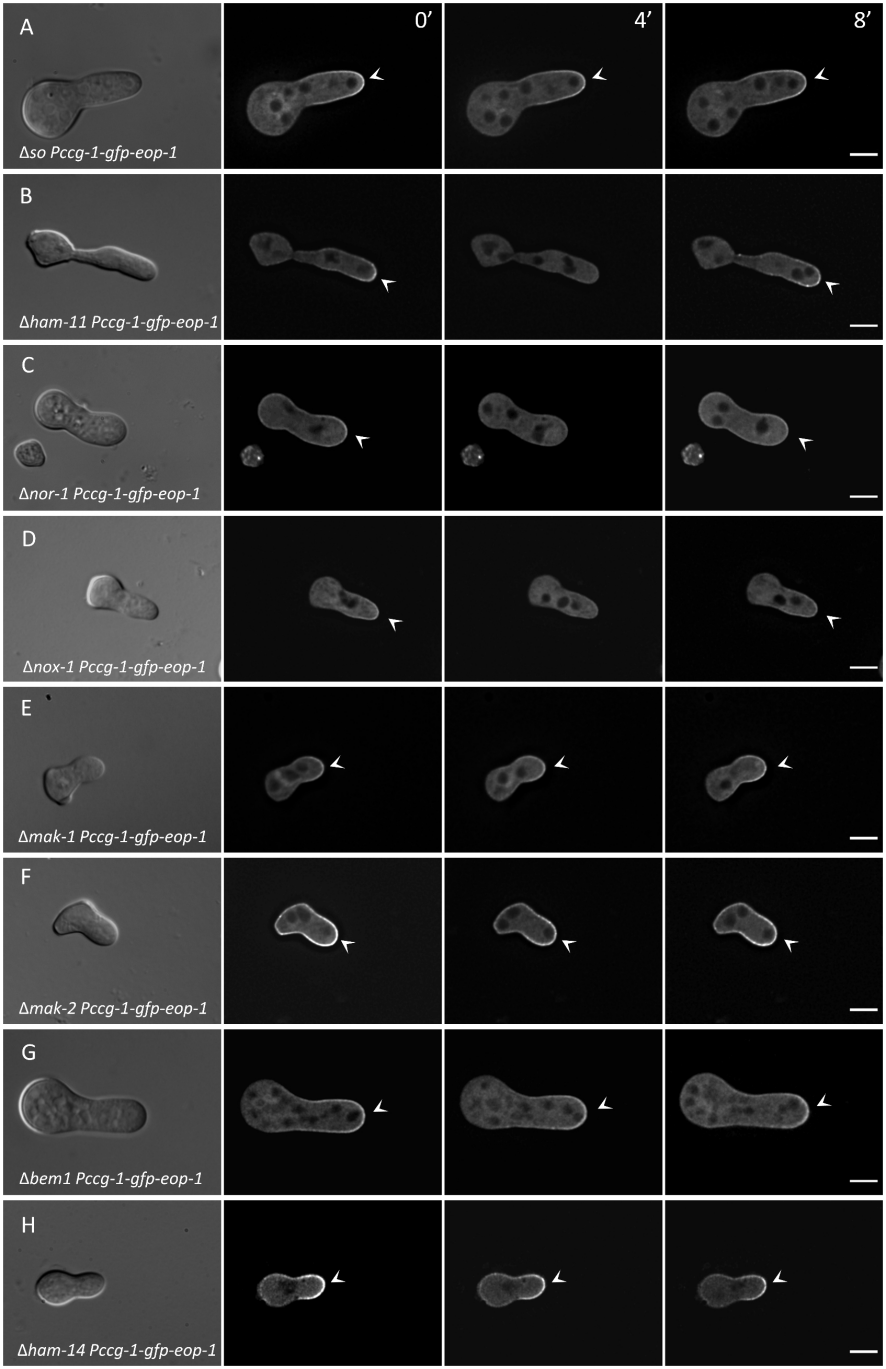
GFP-EOP-1 recruitment patterns in signaling mutants. Deletion of *so*, *mak-1*, *mak-2*, *bem1*, or *ham-14* caused constitutive localization of GFP-EOP-1 at germling tips. In contrast, strains lacking *ham-11*, *nor-1*, or *nox-1* retained the normal oscillatory recruitment pattern of GFP-EOP-1 to the tip. Arrowheads indicate sites of GFP-EOP-1 recruitment. Timepoints 0/4/8 minutes. All scale bars: 5 µm.

We demonstrated that EOP-1 interacts with SO and, unlike other known fusion-related proteins, is recruited to the tips of individual germlings prior to the establishment of interaction. This suggests that EOP-1 may function upstream of SO. To determine whether GFP-EOP-1 membrane recruitment depends on SO, we localized GFP-EOP-1 in a *so* gene deletion strain (GN8–72). In the absence of SO, GFP-EOP-1 was permanently recruited to the tip and did not oscillate (Fig 8A). This result indicates that SO is dispensable for EOP-1 membrane recruitment but is required for its normal spatial dynamics.

The physical interaction between SO and EOP-1 and the dependence of EOP-1 dynamics on SO raised the question of whether SO recruitment is, in turn, affected by the absence of EOP-1. Deletion mutants lacking EOP-1 are unable to undergo cell–cell interactions and therefore cannot reach a stage at which SO recruitment would normally become visible. Consistent with this expectation, we did not detect any recruitment of SO to the plasma membrane in the Δ*eop-1 so-gfp* strain, neither in isolated non-interacting germlings, in germlings in close proximity, nor in hyphae from the colony center or periphery (S2 Fig). Similarly, testing the hierarchy of different fusion related factors had so far been hindered in genetic approaches, since in the gene knockout mutants, cell-cell interactions are completely blocked, preventing the analysis of the subcellular dynamics of other factors at this stage. With EOP-1 as the first factor that oscillates at the membrane before cell-cell interaction, these investigations became now possible.

We examined GFP-EOP-1 localization in strains lacking various communication proteins, including the MAP kinases MAK-1 and MAK-2, the NADPH oxidase complex-related proteins NOX-1 (NCU02110) and NOR-1 (NCU07850), the polarity factor BEM1 (NCU06593), the interaction initiation factor HAM-11 (NCU04732), and HAM-14 (NCU07238), which is specifically involved in germling fusion but not hyphal fusion.

Strains lacking HAM-11 (Fig 8B), NOR-1 (Fig 8C), or NOX-1 (Fig 8D) showed GFP-EOP-1 recruitment to the cell tip in the typical oscillating pattern. In contrast, deletion of MAK-1 (Fig 8E), MAK-2 (Fig 8F), BEM1 (Fig 8G), or HAM-14 (Fig 8H), similar to SO, resulted in permanent recruitment of GFP-EOP-1 to the plasma membrane at the germling tip. These findings suggest that MAK-1, MAK-2, BEM1, and HAM-14 are essential for the dynamic oscillation of EOP-1 in the absence of a communication partner.

### EOP-1 is required for activation of the MAK-1 MAP kinase

The MAK-1 and the MAK-2 MAP kinase pathways are both essential for the tropic interaction of fusing germlings. To determine whether EOP-1 influences their activation, phosphorylation levels of both kinases were analyzed in the Δ*eop-1* mutant and wild-type strains using Phospho-Western Blot analysis (S3 Fig). The results revealed a differential phosphorylation pattern: while MAK-2 phosphorylation remained unaffected, MAK-1 showed little to no phosphorylation in the Δ*eop-1* mutant, indicating significantly reduced activation compared to the wild type.

### C-terminally tagged EOP-1 exhibits an altered recruitment pattern

During the construction of strains for localization experiments, it was observed that strains with C-terminal tags only partially complemented the phenotype typically associated with fusion mutants, as described above. This prompted a detailed investigation of macroscopic growth and microscopic germling interactions in ∆*eop-1*, ∆*eop-1 Peop-1-eop-1-gfp* and ∆*eop-1 Pccg-1-eop-1-gfp*, and wild-type strains (Figs 2 and 3). Comparing aerial hyphae growth revealed that the deletion mutant phenotype could not be fully rescued by the C-terminally tagged EOP-1 variants (Fig 2). While the ∆*eop-1* strain showed severe reductions in aerial hyphae, growth rate, and sporulation, the ∆*eop-1 Peop-1-eop-1-gfp* and ∆*eop-1 Pccg-1-eop-1-gfp* strains exhibited a less pronounced but still significant reduction in aerial hyphae and growth rate relative to the wild type. Germling interaction assays demonstrated that germination rates for ∆*eop-1 Peop-1-eop-1-gfp* (97.0%) and ∆*eop-1 Pccg-1-eop-1-gfp* (100.0%) were comparable to the wild type, yet their interaction rates were significantly reduced (Fig 3A).

In addition to reduced interaction rates, deviations from typical wild-type interaction behavior were observed in interacting germlings. These deviations were classified into three types: 1) formation of multiple interacting cell tips (during chemotropic growth), 2) multiple contacts or cell tips at the contact site, and 3) thickened contact sites. The ∆*eop-1 Peop-1-eop-1-gfp* and ∆*eop-1 Pccg-1-eop-1-gfp* mutants showed a higher frequency of all three deviations compared to the wild type. In contrast, no significant differences were observed between the wild-type strain and the ∆*eop-1 Peop-1-gfp-eop-1* and ∆*eop-1 Pccg-1-gfp-eop-1* strains. (Fig 3D)

**Fig 9:**
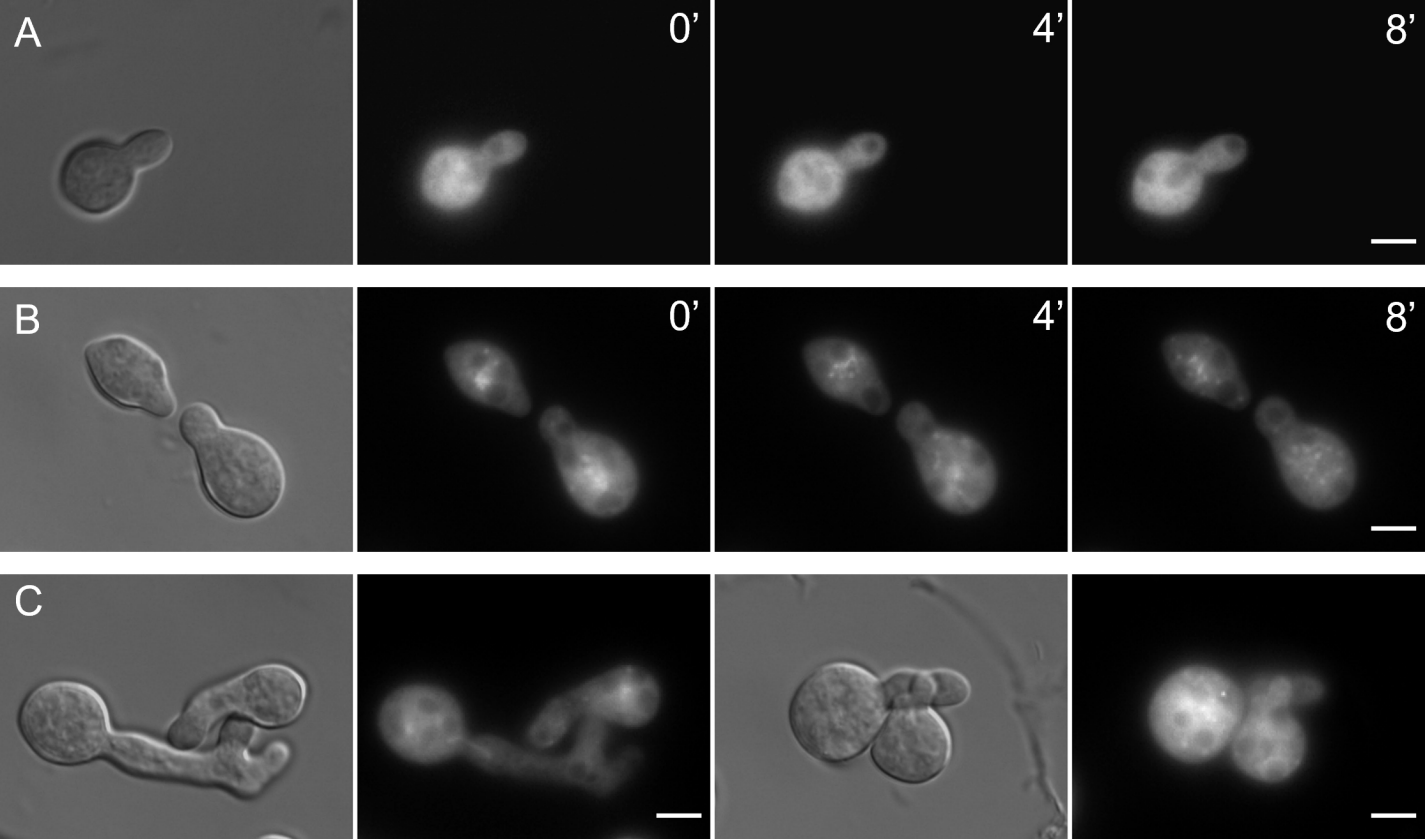
Mislocalization of EOP-1-GFP in germlings. **(A)** In single germlings without partners, EOP-1-GFP is exclusively cytoplasmic, with no membrane recruitment (n = 7). **(B)** In interacting germlings, EOP-1-GFP remains cytoplasmic, showing no tip or membrane recruitment (n = 7). **(C)** After contact, EOP-1-GFP fails to localize at the contact point, remaining cytoplasmic. Timepoints 0/4/8 minutes (n = 7). All scale bars: 5 µm.

These observations indicate that C-terminal GFP tagging disrupts the protein’s function. This prompted an investigation into whether the protein’s localization is similarly affected during interaction and contact. Fluorescence microscopy showed that in individual germlings lacking an interaction partner, EOP-1-GFP localized exclusively to the cytoplasm and was absent from the membrane (Fig 9A). Even in interacting germlings, membrane recruitment was not observed (Fig 9B). Furthermore, after contact between interacting germlings, EOP-1-GFP failed to localize to the contact point as typically seen (Fig 9C). Instead, it consistently remained in the cytoplasm. This mislocalization likely contributes to the impaired complementation of the fusion phenotype, linking the altered localization to the protein’s functional disruption.

## Discussion

Somatic germling and hyphal fusion in *Neurospora crassa* is guided by chemotropic signaling and coordinated by an intricate signaling network including as key factors the fungus-specific SO protein and the MAK-2 MAP kinase module. The current working hypothesis states that the two interacting cells alternate between signal sending associated with the membrane recruitment of SO and signal reception linked to MAK-2 recruitment [14]. This alternation is finely tuned between partner cells and crucial for preventing auto-excitation, ensuring that cells engage in controlled communication and fusion. Until now, the initiation of this interaction was not understood. However, the discovery of EOP-1 as an interaction partner of SO adds a new dimension to our understanding, by suggesting a potential role in the initiation of interaction.

Deletion of *eop-*1 results in a pleiotropic phenotype, impairing not only germling and hyphal fusion but also vegetative growth, aerial hyphae formation, sporulation, and fruiting body development. This broad range of defects is reminiscent of phenotypes seen in other fusion mutants, including the *so* deletion mutant. Similar phenotypes have been reported in deletion mutants of SO homologs in other fungi, such as *Sordaria macrospora* (PRO40), *Podospora anserina* (PaSO), and *Fusarium verticillioides* (FgSO), all of which display defects in both fusion and fruiting body formation [12,20,21]. These pleiotropic phenotypes may result partly from altered colony architecture due to insufficient cross-connectivity via hyphal fusion, but they may also reflect fusion-independent molecular functions of the respective signaling factors [1,22]. These phenotypes are shared with other mutants affected in components of the central signaling network consisting of the MAP kinase cascades, the STRIPAK signaling complex, and two NADPH oxidase complexes [1,8,18,23]. Our findings indicate that EOP-1, as an interaction partner of SO, is also embedded in this network, as reflected by the similar pleiotropic phenotype observed for the deletion mutant.

In contrast to all other known signaling factors taking part in the oscillatory membrane recruitment during cell-cell interaction in *N. crassa*, GFP-EOP-1 exhibits oscillation at the membrane even before any interaction with a partner cell has been initiated, indicating the existence of a cell-autonomous rhythm in germinating spores. When a second germling grows into close proximity, these oscillations become coordinated between the two cells. Only then SO and the MAK-2 MAP kinase module join their specific membrane-associated signaling complexes, indicating successful coordination of the cellular behavior.

The original model proposed that individual germlings continuously release low levels of a signaling molecule even before any interaction occurs. When two germlings grow in close proximity, their combined signal surpasses a threshold, triggering the onset of an oscillatory, pulse-like signal secretion [15]. In contrast, the oscillatory recruitment of EOP-1 prior to any cell-cell interaction suggests that even the initial signal may already be released in a pulsed rather than continuous manner. In this revised view, a signal threshold is still reached when germlings approach each other, but the already pulsatile signal emission becomes coordinated between the cells through entrainment. This coordination then involves the initiation and reinforcement of MAK-2 and SO recruitment to the membrane in the characteristic oscillatory fashion. Our data indicate that oscillatory tip recruitment of EOP-1 marks fusion-competent cells and is not observed in generally polar-growing hyphae within mature colonies. It remains unclear at which stage during early colony establishment hyphae differentiate into the distinct morphological and functional types characteristic of mature colonies, including leading hyphae and lateral branches (both fusion-competent branches and those contributing to further colony expansion). Based on our observations, tip-localized EOP-1 may provide a useful cellular marker for future studies aimed at resolving the timing and mechanisms underlying hyphal differentiation.

Recent work on *Arthrobotrys flagrans* linked oscillatory signaling to pulsating tip growth [24]. In that system, oscillations of the SO and MAK-2 homologous proteins (SofT and MakB, respectively) occur synchronous and in phase with growth oscillations in fusion competent but not yet interacting hyphae. After detection of a fusion partner these oscillations slow and transition into anti-phasic synchronization between the two cells. This has led to the hypothesis that the same oscillatory signaling mechanism operates during both individual growth (“monolog”) and cell-cell communication (“dialog”). In *N. crassa* germlings, however, this type of growth-pulse coupling has not been detected, and SO and MAK-2 are absent from the tips of individual, non-interacting germlings or hyphae. Notably, in this fungus the composition and regulation of the cell polarity machinery differ between interacting and non-interacting cells. The small Rho-type GTPase CDC42 (NCU06454) is both essential and sufficient to drive polarized germ tube growth in non-interacting cells. In contrast, the related GTPase RAC-1 (NCU02160) is specifically required for directed growth toward a partner cell and for successful cell fusion [25]. Thus, distinct Rho-GTPases control different growth behaviors. Together, these findings indicate that the molecular complexes assembled at the cell tip are not identical during individual growth and during cell-cell interaction. Instead, the polarity machinery appears to be reconfigured depending on the cellular context. This contrasts with *A. flagrans*, where our data suggest that similar tip-associated protein assemblies may operate during both individual growth with pre-interaction signal emission and during cell-cell interaction. This indicates that the conserved signaling machinery is wired somewhat differently at distinct stages of the fusion process in these two fungi.

The study of Wernet et al. (2023) [24] offered valuable insights by expanding the coupled oscillator model originally proposed by Goryachev et al. (2012) [15] by explicitly including the concentration of signaling molecules in the extracellular space between hyphae. It is worth exploring whether the mathematical model based on excitable behavior remains applicable to *N. crassa* or if it requires adaptation, to better account for the continuous oscillations observed in cells, spanning from single-cell behavior to interaction and even post-fusion.

In the past, it was a challenge to determine the relationship and hierarchy of the signaling proteins known to be involved in cell fusion. Deletion mutants of fusion-related proteins are typically unable to initiate interaction and do not respond to the presence of potential partner cells. Moreover, the oscillatory recruitment of these proteins only becomes detectable after the interaction between partner cells has already begun. This limitation has hindered efforts to determine the functional hierarchy of the specific factors, as the mutants often fail to reach the developmental stage necessary for such observations. The recruitment of GFP-EOP-1 in non-interaction cells provided a new opportunity to address this question.

Earlier studies suggested a role for the two MAP kinases MAK-1 and MAK-2 in fusion competence. Consistent with this idea, EOP-1 dynamics in individual germlings were disrupted in the absence of these kinases and the protein permanently localized to the plasma membrane. A similar aberrant localization of EOP-1 was observed in the *so* background. Previously, SO was thought to function only after communication between two cells had already been established, since its recruitment to the membrane is detectable only during cell-cell interaction and, the *so* deletion mutant still forms cellular protrusions reminiscent of cell fusion tips [11,14]. The mislocalization of EOP-1 in Δ*so* indicates that SO plays a much earlier role in the interaction process than previously assumed and it is needed for the correct localization of EOP-1 even before its own recruitment to the membrane becomes detectable. Similarly, the absence of the hyphal anastomosis protein HAM-14 [6] and the general polarity factor BEM1 [26] also lead to permanent recruitment of GFP-EOP-1 to the tip of non-interacting germlings, similar to the deletion of SO, suggesting early functions of these proteins, too. In contrast, the proteins HAM-11, NOR-1, and NOX-1 are not required for the autonomous oscillation in single germlings. Although previous hypotheses proposed that HAM-11 plays a role at the very onset of communication, possibly by establishing fusion competence by being involved in the secretion of a competency factor [27], our findings indicate that HAM-11 is not necessary to maintain the cell-autonomous oscillations. NOR-1 and NOX-1 are hypothesized to be important for coordinating cytoskeletal dynamics with other signaling pathways [23] and therefore might be required only later in the communication process during directed growth.

A strain expressing the C-terminally GFP-tagged EOP-1 variant exhibited a phenotype characterized by protein mislocalization in both individual and interacting germlings, reduced interaction frequency, and defective contact recognition. After physical contact, germlings failed to arrest growth, resulting in thickened contact sites and the formation of multiple growing tips that continued to coil around one another.

A similar phenotype has been described for ∆*erg-2* (NCU04156) mutants, strains with reduced *so* expression, and isolates in which MAK-1 activity was chemically inhibited [13]. In ∆*erg-2* mutants, cells fail to enter the growth arrest phase after establishing contact and continue to grow, resulting in the two germ tubes coiling around each other. These mutants accumulate a specific ergosterol precursor that interferes with the correct recruitment of MAK-1 to the plasma membrane at the cell-cell contact point. In addition, SO mislocalizes and also fails to accumulate at the contact zone. When *so* expression is downregulated, a phenotype similar to that of the ∆*erg-2* mutant is observed. Given that SO serves as a scaffold protein within the MAK-1 pathway, its mislocalization likely contributes to the defective recruitment of MAK-1 observed in both mutants. Collectively, these findings suggest that the MAK-1 MAPK pathway plays a central role in regulating contact recognition and growth arrest during germling fusion.

Phosphorylation levels of MAK-1 were significantly reduced in the EOP-1 deletion mutant (S3 Fig), suggesting that EOP-1 contributes to the robust activation of MAK-1 during contact recognition and probably cell wall remodeling during fusion pore formation. These findings support a model in which SO, MAK-1 and EOP-1 function together at the site of cell contact to mediate accurate recognition. This hypothesis is supported by findings in *Podospoa anserina*, where the EOP-1 homologous IDC4 also associates with the cell wall integrity MAP kinase pathway [28].

Analysis of the C-terminally tagged EOP-1 variant (**Fig 3**) indicates that specifically the AIM24-like domain is essential for the process of contact recognition. This domain is conserved across fungi, plants, and bacteria, and has been implicated in functions such as ER stress tolerance in plants or respiratory chain stability in *S. cerevisiae* [28–32]. The role of *N. crassa* EOP-1 in germling and hyphal fusion therefore provides a valuable system to unravel the molecular mechanism underlying the function of this conserved domain.

## Materials and methods

### Strains and growth conditions

The strains used in this study are listed in S3 Table. Cultures were grown on Vogel’s minimal medium (VMM) slants, incubated at 30°C in darkness for 2 days, followed by 3 days of growth at room temperature under a day-night cycle. Genetic methods and media preparation were performed according to protocols provided by the Fungal Genetic Stock Center (www.fgsc.net).

Fusion proteins were expressed at the *his-3* locus under the control of either the *ccg-1* promoter or the native *eop-1* promoter, defined as 1000 bp upstream of the gene. Strains expressing *gfp-eop-1* in knockout backgrounds of other fusion-related genes were generated by crossing the *Pccg1-gfp-eop-1* strain with the respective knockout mutants. Alternatively, *his3-* strains of the respective knockout mutants were created followed by transformation with plasmid 28. Additional strains were constructed as describes below.

### Construction of plasmids and *N. crassa* mutants

For the expression of *gfp-eop-1* under the control of the *ccg1* promotor, the open reading frame of *eop-1* was amplified using Primer 957 and 958 (S4 Table) and cloned into the plasmid pMF334 using restriction sites XbaI and BglII, resulting in plasmid 28. Strain GN8_12 was transformed with plasmid 28 to generate strain GN8_11.

For the expression of *gfp-eop-1* under the control of the native *eop-1* promotor, primers 1058 and 2190 were used to amplify *eop-1* and the *eop-1* promotor. The fragment was cloned into the plasmid pMF334 using ApaI and NotI restriction sites, resulting in plasmid 1063. Strain GN8_12 was transformed with plasmid 1063 to generate strain GN15_47.

For the expression of *eop-1-gfp* under the control of the *ccg1* promotor, *eop-1* was amplified using primer 939 and 940 and cloned into plasmid pMF272 using restriction sites PacI and XbaI, resulting in plasmid 1083. Stain GN8_12 was transformed with the resulting plasmid to generate strain GN16_39.

For the expression of *eop-1-gfp* under the control of the native *eop-1* promotor, primers 1057 and 2593 were used to amplify *eop-1* and the *eop-1* promotor. The fragment was cloned into the plasmid 1083 using NotI and XbaI restriction sites, resulting in plasmid 1244. Strain GN8_12 was transformed with plasmid 1244 to generate strain GN17–66.

For the simultaneous expression of *eop-1* under the control of the *ccg1* promoter and *so* under the control of the *gpd* promoter, plasmid 502 was linearized with XbaI and EcoRI. The *Pccg1-eop-1* insert was excised from plasmid 1243 using XbaI and EcoRI and subsequently ligated into the linearized backbone to create plasmid 1246. Strain GN6–17was transformed with plasmid 1246 to create strain GN17_72.

For the expression of *so-gfp* in the knockout background of *eop-1* strain ∆*so so-gfp* (GN6_52) was crossed with strain Δ*eop-1 his3* (GN8_12). Strain GN6_52 was constructed by transformation of strain GN6_51 with plasmid pSO8 [33].

### Protein structure prediction

The predicted 3D structure of EOP-1, corresponding to the UniProt accession Q1K6G9 was obtained from the AlphaFold Protein Structure Database (https://alphafold.ebi.ac.uk/entry/Q1K6G9) on 21.07.2025.

### Co-immunoprecipitation

For Co-immunoprecipitation (CoIP) followed by MS experiments, spores of the strains *Pccg1-so-gfp* or ∆*eop-1 Pccg-1-eop-1-gfp* respectively were transferred to four 500 mL Erlenmeyer flasks, each containing 100 mL liquid VMM. The cultures were incubated for 4 hours at 30°C without shaking to allow spore germination and interaction. Germlings were harvested by filtering and flash-frozen in liquid nitrogen. Proteins were extracted by adding 500 μL CHAPS buffer, supplemented with 10 μL of 100 mM PMSF, 2.6 μL of 0.5 M benzamidine, and 2 μL of protease inhibitor cocktail IV (Calbiochem). Glass beads (<1 mm) were added, and the samples were lysed in a bead beater for 3 x 30 seconds at 6,500 m/s. Lysates were centrifuged at 13,000 rpm for 30 minutes at 4°C. The 50 µl of the protein supernatant was transferred to a new tube. The protein extract was added to GFP-beads (GFP-Trap_A, Chromotek) and incubated for 3 hours at 4°C on a rotator. After binding, the beads were washed three times with CHAPS buffer, with a final wash supernatant saved as a control.

### In-gel digest and protein purification

For mass spectrometric analysis, SDS-gels were fixed with 30% ethanol/10% acetic acid. Full lanes were cut and protein sample preparation was performed according to [34] with slight changes: the SDS gel was washed with 50mM ammonium bicarbonate (pH 7.8)/40% acetonitrile, alkylation and reduction was performed with 10 mM Tris(2-carboxyethyl)phosphin (TCEP) and 50 mM methyl methanethiosulfonate (MMTS), respectively. After trypsin digestion overnight, the peptides were extracted using 0.2% Trifluoroacetic acid (TFA)/H_2_O before 0.2% TFA/40% acetonitrile.

The extracted samples were dried and resuspended in 0.2% TFA/H2O. Desalting was performed with C18 StageTips as it was described before by Rappsilber et al. (2007) [35], whereas a small amount of Reprosil RP18 10 µm Material was added on top of the Empore disks. The samples were dried again and resuspended in 0.1% Formic acid (FA)/H_2_O for LC-MS.

### LC-MS

Samples were analysed with a Dionex UltiMate 3000 n-RSLC system (Thermo Fisher Scientific), coupled to an Orbitrap Fusion Tribrid mass spectrometer (Thermo Fisher Scientific). All peptide samples were loaded to a C18 precolumn (Acclaim PepMap 100, 75µm x 2 cm, 3 µm particles) for washing before transferring to an analytical column (Acclaim PepMap RSLC, 75µm x 50 cm, 2 µm particles). The peptides were separated in a non-linear gradient from 100% Buffer A (0.1% FA in water) to 70% Buffer B (0.1% FA in 80% Aetonitrile) over 55 minutes with a flow rate of 300 nl/minute or over 90 minutes (0–90% Buffer B) with a flow rate of 200 nl/minute.

Overview spectra were acquired in an Orbitrap scan with a 120K resolution m/z scan window was 350–1500. Precursor ions were collected in a cycle time of 3 s with a AGC target of 400.000 and maximum injection time of 50 ms. Selected ions for fragment spectra were collected with an AGC target of 10.000 and a maximum injection time of 50 ms. The spectra were generated in an ion trap with a 120K resolution after fragmentation by CID with nCE of 35%. The m/z window was set to 350–1500.

### Proteome discoverer and analysis

Peptide identification from MS-raw files was performed with Proteome discoverer (v2.3.0.523) using a Mascot search algorithm (v2.4.2). Enzyme digestion was set to trypsin. A Precursor Mass Tolerance was set to 20 ppm, while the Fragment Mass Tolerance was 0.5 Da. As modifications, a static beta-methylthiolation on cysteines (+45.988), a variable oxidation on methionines (+15.999) and a variable acetylation of N-termini (+42.01) were enabled. The identified peptides were compared to the UniProt database of *N. crassa*. Peptide amounts were quantified according to the precursor ions and normalized according to the total protein amount. Resulting tables were filtered for stably identified proteins with high FDR confidence and at least three unique peptides. For fold-change calculation, missing values were replaced with random small values at the lower identification limit.

### Yeast two hybrid assays

Yeast Two-Hybrid (Y2H) assays were performed in *Saccharomyces cerevisiae* to detect protein-protein interactions using the Gal4 transcription factor system. cDNA of *eop-1* was amplified using primers 1019 and 1020 and cDNA of *so* was amplified using primers 253 and 254. The cDNA of one interaction partner was cloned into pGADT7 (prey, activation domain), and the cDNA of the other was cloned into pGBKT7 (bait, DNA-binding domain) using EcoRI and NdeI restriction sites. Constructs were transformed into *Escherichia coli*, followed by plasmid isolation. The pGADT7 plasmid was transformed into yeast strain AH109 (MATα), and pGBKT7 into Y187 (MATa), with selection on SD-Leu and SD-Trp media, respectively.

Colonies were grown overnight at 30°C in liquid SD-Leu or SD-Trp. Equal cell numbers of both strains were mixed, spread onto YPD + Ade plates, and incubated overnight at 30°C for mating. Diploid cells were verified microscopically. Cell suspensions were plated on SD-Trp-Leu (growth control) and SD-Trp-Leu-Ade-His (interaction selection) plates. Interaction between proteins reconstituted Gal4 activity, enabling growth on selective medium. To confirm specificity, controls with empty vectors were included. Strains with AD-fusion proteins were crossed with strains carrying an empty BD vector, and strains with BD-fusion proteins were crossed with strains carrying an empty AD vector. Media for yeast two-hybrid assays were prepared according to the Matchmaker GAL4 Two-Hybrid System 3 & Libraries User Manual (Clontech Laboratories) [36].

### Macroscopic assays

Growth rate was measured using 45 cm-long glass racetubes filled with 13 ml of Vogel’s MM. Tubes were inoculated with 1x10^5^ spores in 10 µl ddH₂O and incubated at 26°C and daylight. Growth fronts were marked after 48 hours and then every 24 hours to calculate daily growth for 3 replicates per strain.

For aerial hyphal growth assays, test tubes with 2 ml liquid VMM and inoculated with 1x10^5^ spores in 100 µL ddH₂O. Tubes were incubated at 30°C and darkness for seven days to avoid sporulation. Aerial hyphal length was then measured in centimeters for 4 replicates per strain.

For sporulation rate assays, test tubes with 3 ml VMM were inoculated with 1x10^5^ spores in 10 µl ddH₂O. Cultures were incubated at 30°C in darkness for four days, followed by three days at room temperature at daylight. After incubation, 1 ml ddH₂O was added to collect the spores. Spore concentration determined using a Thoma counting chamber for 4 replicates per strain.

### Fluorescence microscopy

To monitor germling interaction, 3 × 10^6^ conidia were spread on VMM plates using a pipette tip. The plates were incubated at 30°C for 4 hours before analysis. For hyphal imaging, 15 µL of conidial suspension was placed at the edge of a Petri dish with VMM and incubated overnight at 30°C in darkness. Observations were made on a Zeiss Observer 2.1 microscope with Nomarski optics and an LED light source (CoolLED pE4000) for fluorescence. A PCO Edge 5.5 Gold (16-bit) camera, controlled by modified 4-D microscopy software by Ralf Schnabel and Christian Hennig, was used for imaging. Basic image analyses were conducted with Fiji (ImageJ), and image stacks were deconvolved using Huygens software (Scientific Volume Imaging, Netherlands).

### Western blot analyses

The phosphorylation status of MAP kinases MAK-1 and MAK-2 was assessed via phospho-specific Western blotting. *N. crassa* wild type (GN6_15) and Δ*eop-1* (GN7_66) strains were cultivated on solid Vogel’s minimal medium for one week and room temperature and daylight. After harvesting, spores were filtered, resuspended in liquid medium, and incubated for 3.5 h without shaking at 30°C to induce germling formation.

Germlings were collected by vacuum filtration and resuspended in protein extraction buffer (PBS with 3 mM KCl, 2.5 mM MgCl₂, 0.1% Tween-20, and 0.5% Triton X-100) supplemented with cOmplete, EDTA-free Protease Inhibitor Cocktail (Roche) and phosphatase inhibitor cocktails II and III (Sigma).

Cells were lysed using a bead beater, with three 10-second cycles at 6000 rpm, followed by centrifugation at 9000 rpm for 10 minutes. All steps post-harvest were conducted on ice or at 4 °C to preserve protein integrity. Supernatants were adjusted to protein concentrations of 5 µg/µl and 10 µg/µl. Electrophoresis, blotting, and antibody detection were carried out as described by Serrano et al. (2018) [19]. For protein loading normalization, membranes were stripped and reprobed with anti-tubulin antibodies. Experiments were performed in triplicate.

### Sexual crossing

To induce protoperithecia formation, one of the mating partners was grown on plates with Westergaard’s medium for approximately 7 days at 26 °C and daylight. [37] To provide sufficient conidia, the second mating partner was incubated as described above. Fertilization of the protoperithecia was achieved by spreading harvested conidia onto the Westergaard’s plates and subsequent incubation at 26°C and daylight. The development was documented photographically 7 days after inoculation and 21 days after fertilization.

### Statistical analysis

Data are presented as mean values ± SD of all biological replicates. *P*-values of two-tailed Student’s *t*-tests or students Fisher’s exact test are provided in the figure legends.

## Supporting information

S1 Table NCU04645 is a potential interaction partner of SO. Results of mass spectrometric analysis of co-precipitated proteins from SO-Co-IP were compared with the respective data of the wash fraction. The list shows exclusively identified proteins in the SO-IP eluate fraction.
(XLSX)

S2 Table List of all identified potential interaction partners of SO and NCU04645, identified by Co-immunoprecipitation experiments followed by mass spectrometry. (XLSX)

S1 Fig Sexual development of the *eop-1* deletion mutant on Westergaard’s medium. (PDF)

S2 Fig Localization of SO-GFP in germlings and hyphae in the strain ∆*eop-1 Pccg-1-so-gfp.* (PDF)

S3 Fig Phospho-Western Blot analysis. (PDF)

S3 Table Strain list. (PDF)

S4 Table Primer list. (PDF)
