# Supplementary material for: Characterization of EOP-1 reveals cell autonomous oscillations preceding somatic cell fusion in Neurospora crassa
Source: PLoS Genet. 2026 Mar 31;22(3):e1012087. doi: 10.1371/journal.pgen.1012087 (PMC13075794; doi:10.1371/journal.pgen.1012087)
Supplement: S1 Fig — (PDF) [file pgen.1012087.s003.pdf]

Figure S3

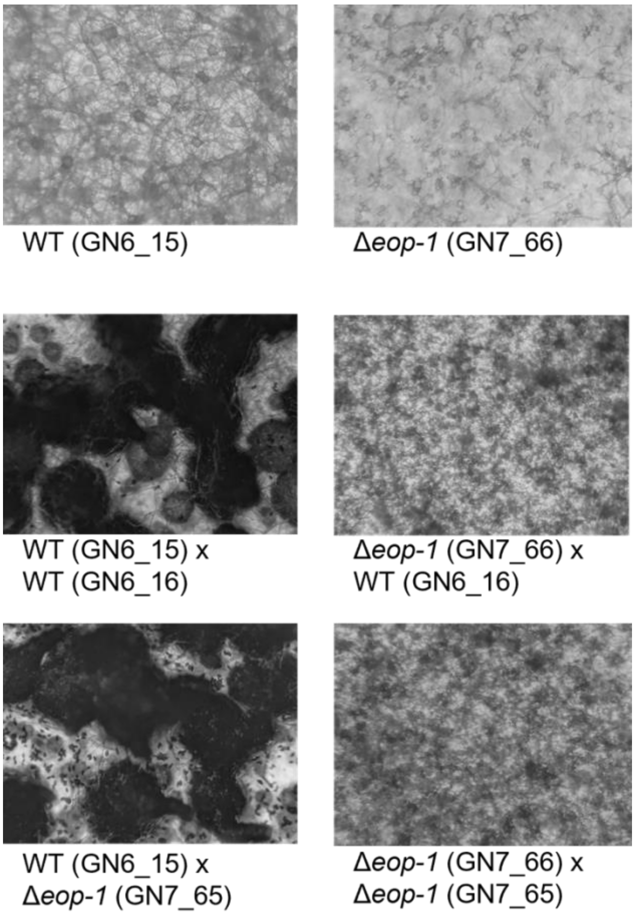

Sexual development of the *eop-1* deletion mutant on Westergard's medium. Protoperithecia formation was assessed after 7 days of incubation on Westergaard's medium at 26°C under day light (n = 3). Δ*eop-1* mutants formed very few protoperithecia while the WT showed normal protoperithecia formation. Fertility tests showed that Δ*eop-1* mutants were sterile as female mating partners but displayed normal fertility when used as males. The strain used as female is listed first.
