# Supplementary material for: Characterization of EOP-1 reveals cell autonomous oscillations preceding somatic cell fusion in Neurospora crassa
Source: PLoS Genet. 2026 Mar 31;22(3):e1012087. doi: 10.1371/journal.pgen.1012087 (PMC13075794; doi:10.1371/journal.pgen.1012087)
Supplement: S2 Fig — (PDF) [file pgen.1012087.s004.pdf]

S4 Figure

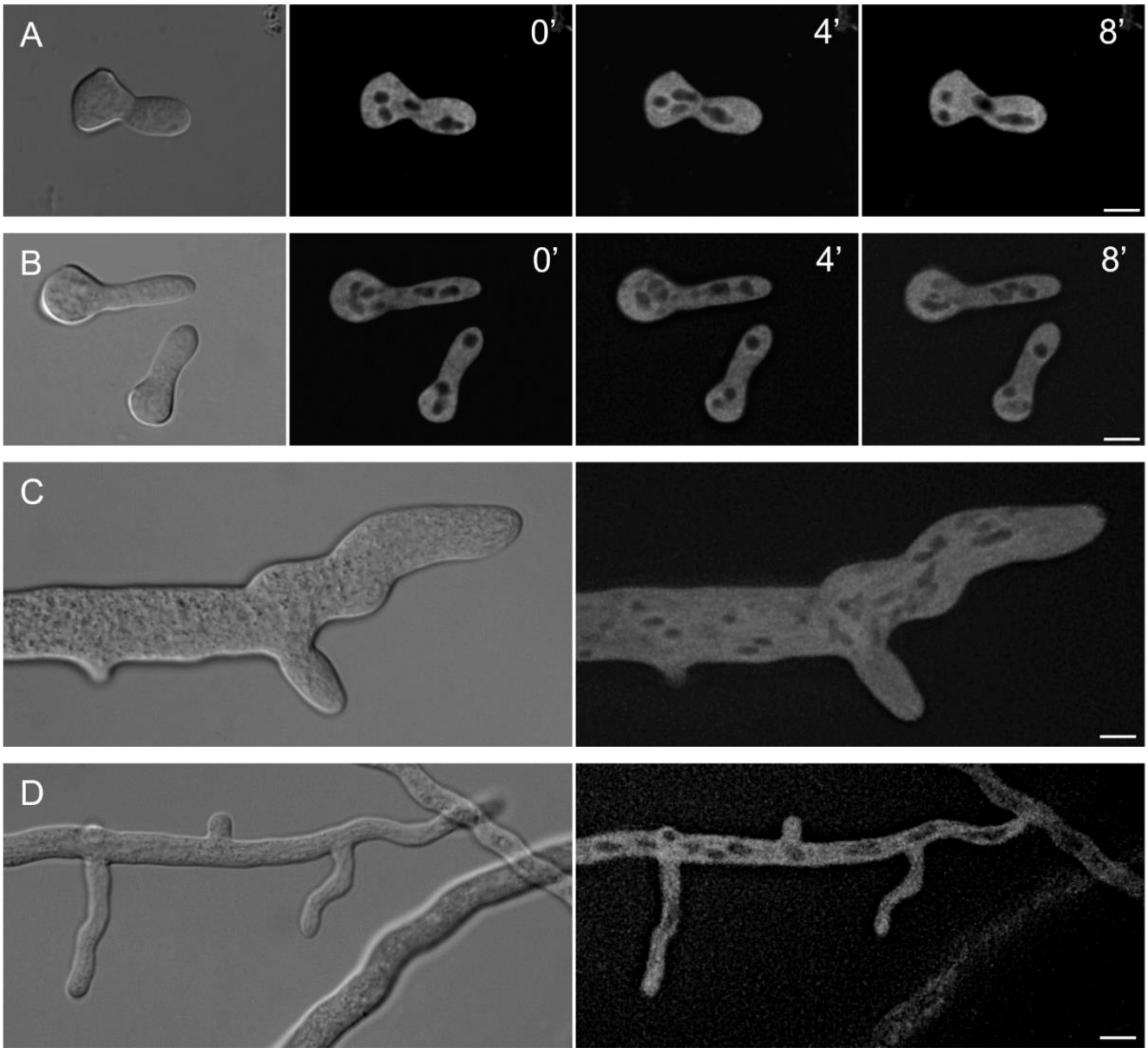

Localization of SO-GFP in germlings and hyphae in the strain Δ*eop-1 Pccg-1-so-gfp*. SO-GFP localizes to the cytoplasm in individual germlings (A) as well as non-interacting germlings in close proximity (B). In hyphal tips from the edge of the colony (C) as well as hyphal tips from inner parts of the colony (C), SO-GFP localizes to the cytoplasm. All scale bars 5 µm.
