# Supplementary material for: Characterization of EOP-1 reveals cell autonomous oscillations preceding somatic cell fusion in Neurospora crassa
Source: PLoS Genet. 2026 Mar 31;22(3):e1012087. doi: 10.1371/journal.pgen.1012087 (PMC13075794; doi:10.1371/journal.pgen.1012087)
Supplement: S3 Fig — (PDF) [file pgen.1012087.s005.pdf]

Figure S5

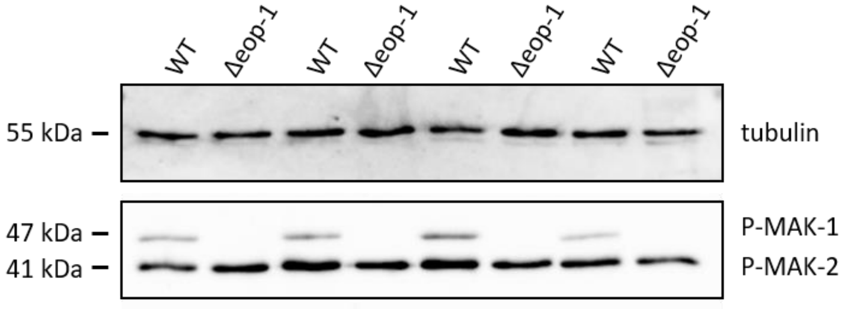

Western blot analysis of total cell extracts from WT and Δ*eop-1* strains in four biological replicates. Blots were probed with antibodies against tubulin (loading control), phosphorylated MAK-1 (P-MAK-1), and phosphorylated MAK-2 (P-MAK-2).
