# Supplementary material for: Characterization of EOP-1 reveals cell autonomous oscillations preceding somatic cell fusion in Neurospora crassa
Source: PLoS Genet. 2026 Mar 31;22(3):e1012087. doi: 10.1371/journal.pgen.1012087 (PMC13075794; doi:10.1371/journal.pgen.1012087)
Supplement: S3 Table — (PDF) [file pgen.1012087.s006.pdf]

S6 Table. Strains used and created in this study

Number	Name	Genotype	Origin
GN6_15	WT	N. crassa *mat*A (wild type)	FGSC 2489
GN6_16	WT	N. crassa *mata* (wild type)	FGSC 988
GN6-17	*his3*	*his3, matA*	FGSC 6103
GN6-18	*his3*	*his3, mata*	FGSC 9716
GN8_12	*Δeop-1 his3*	*eop-1::hph his3 matA*	this study
GN8_11	*Pccg1-gpf-eop-1*	*eop-1::hph, his3::Pccg1-gfp-eop-1, matA*	this study
N1-20	*Pccg1-so-gfp*	*his3::Pccg1-so-gfp, matA*	(Fleissner und Glass 2007)
GN7_66	*Δeop-1*	*eop-1::hph, matA*	this study
GN7-65	*Δeop-1*	*eop-1::hph, mata*	FGSC 19133
GN8_12	*Δeop-1 his3*	*eop-1::hph his3 matA*	this study
GN15_47	*Peop-1-gpf-eop*	*eop-1::hph, his3::Peop1-gfp-eop-1, matA*	this study
GN8_11	*Pccg1-gpf-eop-1*	*eop-1::hph, his3::Pccg1-gfp-eop-1, matA*	this study
GN17_66	*Peop-1-eop-1-eop-1*	*eop-1::hph, his3::Peop-1-eop-1-gfp, matA*	this study
GN16_39	*Pccg1-eop-1-gfp*	*eop-1::hph, his3::Pccg1-eop-1-gfp, matA*	this study
GN17-72	*gfp-eop-1, so-dsRed*	*his3::Pccg1-gfp-eop-1, Pgpd-so-dsred, matA*	this study
GN8_33	*Δeop-1 so-gfp*	*eop-1::hph, his3::Pccg1-so-gfp*	this study
GN9_12	*Δmak-2 gfp-eop-1*	*mak-2::hph, his3::Pccg1-gfp-eop-1*	this study
GN9-23	*Δmak-2 his3*	*mak-2::hph, his3, al-1, matA, DIP*	(Fleißner et al. 2009)
GN8-72	*Δso Pccg1-gfp-eop-1*	*so::hph his3::Pccg1-gfp-eop-1*	this study
GN6-51	*Δso his-*	*so::hph his3; mata*	(Weichert et al. 2016)
GN6-52	*Δso so-gfp*	*so::hph his3::Pccg1-so-gfp, mata*	this study
AO_028	*Δmak-1 Pccg1-gfp-eop-1*	*mak-1::hph, his3::Pccg1-gfp-eop-1, matA*	this study
MW_486	*Δmak-1 his3*	*mak-1::hph, his3, matA*	(Weichert et al. 2016)
AO_100	*Δham-14 Pccg1-gfp-eop-1*	*ham-14::hph, his3::Pccg1-gfp-eop-1, mata*	this study
KO-0357	*Δham-14*	*Δham-14, mata*	FGSC 14336
2-A1	*Δbem-1 his-3- Δmus-52*	*bem::hph; mus-52::bar+; his3; mat A*	(Schürg et al. 2012)
AO_027	*Δbem-1 Pccg1-gfp-eop-1*	*bem::hph; gfp-sip-1; Δmus-52::bar+; mat A*	this study
AO_026	*Δham-11 Pccg1-gfp-eop-1*	*ham-11::hph, his3::Pccg1-gfp-eop-1, mata*	this study
KO-0489	*Δham-11*	*ham-11::hph, matA*	FGSC 17545
AO_012	*Δham-11 his3*	*ham-11::hph his3 mata*	this study
AO_015	*Δnor-1 Pccg1-gfp-eop-1*	*nor-1::hph, his3::Pccg1-gfp-eop-1, matA*	this study
AS-55-724	*Δnor-1 his3*	*nor-1::hph his3*	(Serrano et al. 2018)
AO_021	*Δnox-1 Pccg1-gfp-eop-1*	*nox-1::hph, eop-1::hph, his3::Pccg1-gfp-eop-1, matA*	this study
KO-0045	*Δnox-1*	*nox-1::hph, mata*	FGSC 12867
