# Supplementary material for: Characterization of EOP-1 reveals cell autonomous oscillations preceding somatic cell fusion in Neurospora crassa
Source: PLoS Genet. 2026 Mar 31;22(3):e1012087. doi: 10.1371/journal.pgen.1012087 (PMC13075794; doi:10.1371/journal.pgen.1012087)
Supplement: S4 Table — (PDF) [file pgen.1012087.s007.pdf]

S7 Table. Oligonucleotides used in this study

ID	Nucleotide sequence (5′–3′)
253	AATGTACATATGTCTCGATCCCGCGGTGTTC
254	AATGTAGAATTCCTAATGCCCATACTCCAAATGC
939	AATGTATCTAGAATGTCTTACTATCCTCGTGAG
940	TACATTTTAATTAAGGCCTTTGCTGTTTGACCG
957	AATGTTCTAGACTAGGCCTTTGCTGTTTGACCGGTC
958	AATGTAAGATCTATGTCTTACTATCCTCGTGAG
1019	ATCATCcatatgTCTTACTATCCTCCACC
1019	ATCATCCATATGTCTTACTATCCTCCACC
1020	ATCATCgaattcCTAGGCCTTTGCTGTTTGAC
1020	ATCATCGAATTCCTAGGCCTTTGCTGTTTGAC
1057	AATGTAGCGGCCGCCTTATCATTCATGGACATTC
1058	AATGTAGGGCCCCTTATCATTCATGGACATTC
2190	GCGGATAACAATTTCACACAGGAAACAGCGCGGCCGCCTCGCAAACTTGGACTTTCTTTC
2593	AATGTATCTAGAATTGATTGTTTGTTTTTCTCGAAC
